# *QuickStats:* Age-Adjusted Rate* of Motor Vehicle Traffic Deaths,^^†^^ by Urbanization of County of Residence^^§^^ — 2005 and 2015

**DOI:** 10.15585/mmwr.mm6621a6

**Published:** 2017-06-02

**Figure:**
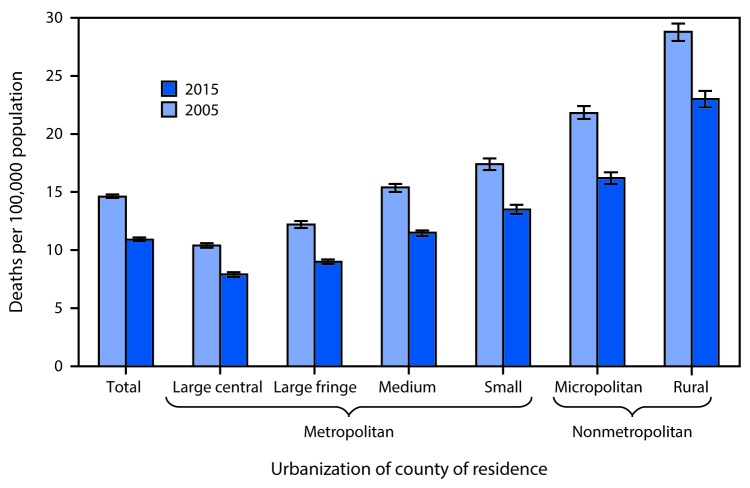
The overall age-adjusted rate of motor vehicle traffic deaths in the United States decreased 25% from 14.6 deaths per 100,000 population in 2005 to 10.9 in 2015. During this period, the rate declined in each of the county groupings, with the largest decline of 26% in the large fringe metropolitan and micropolitan counties and the smallest decline of 20% in rural counties. For both years, the rates for motor vehicle traffic deaths were higher in nonmetropolitan areas than in metropolitan areas. In 2015, the age-adjusted rate in rural counties was nearly three times the rate for large central metropolitan counties (23.0 compared with 7.9 per 100,000).

**Sources:** National Center for Health Statistics, National Vital Statistics System, Mortality File https://www.cdc.gov/nchs/nvss/deaths.htm.

Ingram DD, Franco SJ. 2013 National Center for Health Statistics urban-rural classification scheme for counties. Vital Health Stat 2014;2(166). https://www.cdc.gov/nchs/data/series/sr_02/sr02_166.pdf.

For more information on this topic, CDC recommends the following link: https://www.cdc.gov/motorvehiclesafety/.

